# A Bioactive Cartilage Graft of IGF1-Transduced Adipose Mesenchymal Stem Cells Embedded in an Alginate/Bovine Cartilage Matrix Tridimensional Scaffold

**DOI:** 10.1155/2019/9792369

**Published:** 2019-04-18

**Authors:** Nidia K. Moncada-Saucedo, Iván A. Marino-Martínez, Jorge Lara-Arias, Víktor J. Romero-Díaz, Alberto Camacho, J. A. Valdés-Franco, Vanessa Pérez-Silos, Alejandro García-Ruiz, Hang Lin, Rocky S. Tuan, Rosalío Ramos-Payán, M. Lara-Banda, Rocio Ortiz-Lopez, Augusto Rojas-Martinez, Lizeth Fuentes-Mera

**Affiliations:** ^1^Universidad Autónoma de Nuevo León (UANL), Departamento de Bioquímica y Medicina Molecular, Facultad de Medicina, Monterrey, NL, Mexico; ^2^Universidad Autónoma de Nuevo León (UANL), Unidad de Terapias Experimentales, Centro de Investigación y Desarrollo en Ciencias de la Salud, Monterrey, NL, Mexico; ^3^Universidad Autónoma de Nuevo León (UANL), Departamento de Patología, Facultad de Medicina, Monterrey, NL, Mexico; ^4^Universidad Autónoma de Nuevo León (UANL), Servicio de Ortopedia y Traumatología, Hospital Universitario “Dr. José E. González”, Monterrey, NL, Mexico; ^5^Universidad Autónoma de Nuevo León (UANL), Departamento de Histología, Facultad de Medicina, Monterrey, NL, Mexico; ^6^Universidad Autónoma de Nuevo León (UANL), Unidad de Neurometabolismo, Centro de Investigación y Desarrollo en Ciencias de la Salud, Monterrey, NL, Mexico; ^7^Universidad Autónoma de Nuevo León, Monterrey, NL, Mexico; ^8^Plant Breeding and Genetics Section, School of Integrative Plant Science, Cornell University, Ithaca, NY, USA; ^9^Department of Orthopaedic Surgery, McGowan Institute for Regenerative Medicine, University of Pittsburgh School of Medicine, Pittsburgh, PA, USA; ^10^Center for Cellular and Molecular Engineering, Department of Orthopaedic Surgery, University of Pittsburgh School of Medicine, Pittsburgh, PA, USA; ^11^Department of Bioengineering, Swanson School of Engineering, University of Pittsburgh, Pittsburgh, PA, USA; ^12^Sciences Faculty, Autonomous University of Culiacan, Culiacan, Sinaloa, Mexico; ^13^Universidad Autónoma de Nuevo León, Facultad de Ingeniería Mecánica y Eléctrica, Monterrey, NL, Mexico; ^14^Tecnologico de Monterrey, Escuela de Medicina y Ciencias de la Salud, Monterrey, NL, Mexico

## Abstract

Articular cartilage injuries remain as a therapeutic challenge due to the limited regeneration potential of this tissue. Cartilage engineering grafts combining chondrogenic cells, scaffold materials, and microenvironmental factors are emerging as promissory alternatives. The design of an adequate scaffold resembling the physicochemical features of natural cartilage and able to support chondrogenesis in the implants is a crucial topic to solve. This study reports the development of an implant constructed with IGF1-transduced adipose-derived mesenchymal stem cells (immunophenotypes: CD105^+^, CD90^+^, CD73^+^, CD14^−^, and CD34^−^) embedded in a scaffold composed of a mix of alginate/milled bovine decellularized knee material which was cultivated *in vitro* for 28 days (3CI). Histological analyses demonstrated the distribution into isogenous groups of chondrocytes surrounded by a de novo dense extracellular matrix with balanced proportions of collagens II and I and high amounts of sulfated proteoglycans which also evidenced adequate cell proliferation and differentiation. This graft also shoved mechanical properties resembling the natural knee cartilage. A modified Bern/O'Driscoll scale showed that the 3CI implants had a significantly higher score than the 2CI implants lacking cells transduced with IGF1 (16/18 vs. 14/18), representing high-quality engineering cartilage suitable for *in vivo* tests. This study suggests that this graft resembles several features of typical hyaline cartilage and will be promissory for preclinical studies for cartilage regeneration.

## 1. Introduction

Injuries of joint cartilage may result in incapacitating damage and increased susceptibility to early osteoarthritis [1]. Cartilage tissue engineering is an emerging, promising approach to repair joint cartilage defects and restore joint function [2, 3]. This approach commonly includes an ex vivo tissue regeneration procedure that involves chondrocytes or mesenchymal stem cells (MSCs) in a three-dimensional, porous biomaterial by a strategy with potential for tissue regeneration [4].

Interest in the construction of genetically engineered functional cartilage as an alternative for cartilage regeneration has been increasing, and it is aimed at providing a fully functional replacement of the damaged tissue. This includes not only the structure but also the biomechanical features of the cartilage tissue [5, 6]. In recent years, several designs based on the use of scaffolds enriched by an extracellular matrix (ECM) of decellularized cartilage to construct tissue-engineered cartilage have been reported [7–10]. The ECM from decellularized cartilage, obtained by controlled protocols, preserves bioactive proteins, which provide a biomimetic microenvironment suitable for cellular adhesion and proliferation [11], potentially improving its biomechanical features.

The ECM is composed of proteoglycans, where the core proteins of proteoglycans are modified by glycosaminoglycans (GAGs) between them, chondroitin sulfate, keratan sulfate, and dermatan sulfate. The GAG side chains also give compressive and osmotic swelling properties to the cartilage by entrapping water [12, 13]. Heparan sulfate has a high affinity with a wide range of growth factors crucial for cartilage homeostasis [14, 15]. Due to this, the presence and distribution of proteoglycans have an intimate influence on the mechanical features of native tissue.

The fact that MSCs can be acquired autologously and their potential for multilineage differentiation and proliferation *in vitro* make MSCs a more attractive source of cells for tissue engineering than adult human chondrocytes [16]. Through appropriate biochemical and mechanical stimuli, MCSs have been shown to generate engineered cartilage that more closely correlates with native hyaline articular cartilage [17, 18].

Chondroinduction of MSCs is influenced by many growth factors, as well as external mechanical stimuli such as three-dimensional (3D) culture in scaffolds, hydrostatic pressure, and dynamic compression [19–21]. Typically, MSCs on scaffolds or hydrogels are directed towards a chondrocytic lineage *in vitro* using the first chondrogenic medium, which are then released, repassaged, and reseeded onto scaffolds and further cultured to obtain mature tissue-engineered cartilage [22]. Several growth factors also play important roles in normal cartilage metabolism [23, 24], where insulin-like growth factor-1 (IGF1) promotes the synthesis of ECM representing an outstanding growth factor for gene therapy of osteoarthritis [25]. We previously analyzed the *in vitro* chondrogenesis of ADSCs transduced with adenoviral vectors encoding IGF1, transforming growth factor beta-1 (TGF*β*1), fibroblast growth factor-2 (FGF2), and sex-determining region Y-box 9 (SOX9), either alone or in combination. The results demonstrated that overexpression of IGF1 and FGF2 or IGF1 alone resulted in high expression levels of collagen II, proteoglycan, aggrecan, biglycan, and cartilage matrix and, hence, a better ECM production under a monolayer system [26].

In the present study, we investigated whether the 3D system consisting of a three-component implant (3CI)—MSCs derived from adipose tissue (ADSCs) transduced with IGF1 and embedded in a scaffold made of bovine cartilage/alginate (BCM/A) matured for 28 days—would drive the generation of a cartilage tissue that resembles the architecture found in native cartilage with adequate mechanical properties.

## 2. Materials and Methods

### 2.1. Ovine Adipose-Derived Stem Cell Isolation and Cell Cultivation

ADSCs were harvested from the adipose tissue of 3-month-old sheep weighing 16-19 kg. To obtain samples of adipose tissue, lipectomy was conducted exclusively within the area of the rib cage. The adipose tissue sample (0.5 g) was minced into small pieces and digested in 0.1% collagenase I (Gibco-Invitrogen, Carlsbad, CA) at 37°C for 2 hr. The cell suspension was filtered through a 100 *μ*m filter (BD Falcon, BD Biosciences, San Jose, CA) for the removal of solid aggregates. The sample was centrifuged at 2000 rpm for 5 min at room temperature, and the supernatant removed without disturbing the cells on the bottom. The pellet was resuspended in DMEM supplemented with 10% fetal bovine serum (FBS) (HyClone, Logan, UT), L-glutamine (10 mM), and a mixture of penicillin (100 U/ml), streptomycin (100 *μ*g/ml), and amphotericin B (0.25 mg/ml) (all from Corning Cellgro, Manassas, VA) and cultured in a 25 cm^2^ flask with a humidified atmosphere of 5% CO_2_ at 37°C. After a week, nonadherent cells were removed and adherent cells were further cultured in supplemented DMEM. The medium was changed every 3 days until the monolayer of adherent cells reached 70-80% confluence.

### 2.2. Flow Cytometry Analysis

The immunophenotype was analyzed within the 4th and 5th passages by fluorescence-activated cell sorting (FACS). In brief, 5 × 10^5^ cells (in 50 *μ*l of staining buffer (SB, Pharmingen Becton Dickinson)) were mixed with 10 *μ*l of the following antibodies: PE-conjugated CD73 (BioLegend, San Diego, CA), FITC-conjugated CD90 (Santa Cruz Biotechnology, California), PE-conjugated CD34 (BD Biosciences, NJ), RPE-conjugated CD45 (BD Biosciences, NJ), RPE-conjugated CD14 (Serotec, Kidlington, UK), and APC-conjugated CD105 (BioLegend). Proper isotype controls for each antibody were used to discard unspecific binding. Cells were incubated for 20 min at room temperature in the dark and then fixed and analyzed using an Attune flow cytometer (Life Technologies, Carlsbad, CA). Data analysis was performed using FlowJo version 7.2.2 software (Tree Star Inc., Ashland, OR). Nonviable cells were excluded according to the side scatter vs. forward scatter parameters, and 5,000 events were acquired for each sample.

### 2.3. Chondrogenic Induction

To induce chondrogenic differentiation, ovine ADSCs were plated at 2 × 10^3^ cells/cm^2^ in chondrogenic medium containing DMEM (Gibco, Scotland, UK) supplemented with 10% FBS, 1% GlutaMAX (Gibco), 1% antibiotic antimycotic (Gibco), 1% vitamin C (Sigma), 1% insulin-transferrin-selenium (Gibco), 50 *μ*g/ml ascorbic acid-2-phosphate (Sigma, USA), 40 ng/ml L-proline (Sigma), and 100 nM dexamethasone (Invitrogen Inc.). Cultures were maintained for 2 weeks, with frequent medium changes every 2–3 days. The prechondrocyte phenotype was controlled by analyzing the expression of the SOX9, Col-II, and RUNX2 markers by RT-PCR for the subsequent viability assay.

### 2.4. Trilineage Differentiation Assays

For adipogenic differentiation, ovine ADSCs were grown to 70% confluence in a 4-well chamber (Thermo Fischer Scientific, Nunc) in the DMEM supplemented with 10% FBS. Adipogenesis was induced with adipogenic medium containing 1 mM dexamethasone, 1 mg/ml insulin, 0.5 mM 3-isobutyl-1-methylxanthine, and 100 mM indomethacin. After 3 weeks, the cultures were fixed in 10% paraformaldehyde and stained with oil red O solution to detect lipid droplets. Induction to chondrogenic lineage is described in Section 2.3.

To induce osteogenic differentiation, ovine ADSCs (2 × 10^3^ cells/cm^2^) were grown in osteogenic medium (DMEM supplemented with 10% FBS, 10 mM *β*-glycerophosphate, 0.25 mM ascorbic acid, and 10-8 M dexamethasone). Cultures were maintained for 3 weeks with medium changes every 3 days, and alizarin red S staining was performed to evaluate calcium-rich deposits. Control cultures of ADSCs without differentiation medium were also maintained simultaneously (uninduced group).

### 2.5. Preparation of Natural Bovine Cartilage Matrix (BCM)

The cartilage matrix was prepared from bovine knees, obtaining approx. 2 mm slabs. For decellularization, tissue pieces were washed with PBS buffer and subjected to 5 cycles of freezing in liquid nitrogen for 5 min and thawed at 37°C in distilled water. Subsequently, washes with PBS for 10 minutes were given. The product was triturated in a blender for 20 min and incubated with 2% SDS for 9 h at 37°C. The slices were washed for 2 h with PBS changes every 30 min. Then, the material was finely grinded in a mill (Micron, Mexico City, Mexico) and sterilized with methylene oxide. Percentage of empty lacunae and MEC integrity were assessed using H&E staining and Masson's trichrome staining.

### 2.6. Implant Construction

In brief, 5 × 10^5^ prechondrocytes (see Section 2.3) were resuspended in a solution of 1% BCM fine powder mixed with 1.2% alginate/DMEM. A sterile alginate solution was prepared by dissolving ultrapure sterile sodium alginate (Pronova UP MVG, NovaMatrix, FMC Biopolymer) in 1.2 wt.% DMEM.

To allow the gelation of the implants, the mixture was dripped (20-40 *μ*l) in a CaCl_2_ solution (0.102 M) and maintained for 15 minutes. The cellularized implants were incubated with chondrogenic medium in a 24-well plate for 14 more days, to reach an *in vitro* maturation period of 28 days, and the medium was changed every three days. Two groups of implants supported on alginate/BCM scaffolds were constructed: the implant with ovine ADSCs transduced with Ad-IGF1 vector (3CI) and control with nontransduced ovine ADSCs (2CI).

### 2.7. Mechanical Testing

Acellular alginate was subjected to unconfined compression tests, and alginate/BCM scaffolds (*n* = 6) were punched into 3 mm diameter circular discs. Mechanical testing of scaffolds was conducted with a mechanical tester (Bose Electroforce model 3230 Series II). The exact dimension of scaffolds was measured with a caliper, and the scaffolds were then placed between the compressive motor and load cell and subjected to 10% compression at 0.01 mm/s. Young's modulus of scaffolds was determined from the slope of force versus displacement plots. All testing groups in this study consisted of 5 individual samples, and the statistical significance between the groups was determined by one-way ANOVA followed by the Tukey-Kramer HSD test. Samples with a *p* < 0.05 were determined to be statistically significant.

### 2.8. Alamar Blue Assay

Viability of ovine ADSCs and prechondrocytes on the scaffolds was measured using the Alamar Blue assay (AB, Invitrogen, USA) on days 3, 5, and 7 postseeding. After initial seeding in DMEM, at each time point, supernatants were removed and 1 ml of fresh medium containing 10% (*v*/*v*) AB was added into each well. After 4 hours, three copies of 100 *μ*l aliquots of AB containing medium were moved into a 96-well dark plate for absorbance measurement at 570 nm. An equal volume of fresh medium without AB was added to each well for the next measurement.

### 2.9. Immunofluorescence from Paraffin-Embedded Sections

For the analysis of cell distribution along the scaffolds (alginate vs. alginate/BCM), immunofluorescence was performed in 10 *μ*m thick paraffin-embedded tissue sections, using a heat-induced antigen retrieval process in a 0.01 mol/L solution of sodium citrate before incubation with DAPI. The coverslips were washed three times with PBS and then in sterile distilled water and allowed to dry before mounting with VECTASHIELD with DAPI (Vector Laboratories, Burlingame, CA). All slides were examined using a DMRA microscope (Leica Microsystems, Wetzlar, Germany) fitted with appropriate fluorescence filters, and the images were captured using CytoVision software (Applied Imaging Systems, Santa Clara, CA).

### 2.10. Scanning Electron Microscopy

After primary fixation in 4% glutaraldehyde, all samples were soaked in 0.1 M sodium cacodylate for 10 minutes and washed three times. Samples were transferred into 1% osmium tetraoxide for two hours for postfixation and washed again with 0.1 M sodium cacodylate. Samples were dehydrated in a graded series of acetone, placed into the critical point dryer (CPD Baltec-030) for 30 minutes, mounted onto a stub sputtered with gold coating in Sputter Coater Polaron E-5100 SEM Coating Unit, and viewed under a JEOL JSM 6400 scanning electron microscope (JEOL Corporation Ltd., Tokyo, Japan).

### 2.11. Adenoviral Vector Transduction with Ad-IGF1

Construction of the replication-deficient viral vector was previously described [26]. The adenovirus E1A region was replaced with the human IGF1 gene under the control of the Rous sarcoma virus (RSV) long-terminal repeat and grown in HEK 293 cells. The transduction assay was performed using the Ad-IGF1 vector with an MOI of 100 from an aliquot of 7.94 × 10^9^ PFU/ml. A total of 1 × 10^6^ cells were seeded in a 25 cm^2^ flask, and the next day, the culture medium was removed and 1 ml of DMEM was added to the amount of viral particles as necessary. Cells were incubated with the viral particles for 3 h at 37°C. Subsequently, 4 ml of DMEM supplemented with 5% FBS was added and incubated for 7 days.

### 2.12. Quantitative IGF1 ELISA

ADSCs and Ad-IGF1 were embedded in the scaffold; the resulting constructs 2CI and 3CI (respectively) were cultured in chondrogenic medium. The concentration of the growth factor IGF1 secreted in 24 h by them was measured quantitatively from culture supernatants collected at 0, 7, and 14 days of culture using ELISA (R&D Systems, Minneapolis, MN, USA). The optical density of each well was determined immediately using a microplate reader (ELISA plate spectrophotometer, Molecular Devices, Sunnyvale, CA, USA). The secreted cytokines were normalized to the quantity of protein measured by the Bradford protein assay. Three independent experiments were done in duplicate for each experimental condition. The final results of the immunoassay were determined by measuring the samples' optical density at 450 nm and using a 540 nm wavelength correction. Additionally, a standard curve was generated using the standard provided in the kit and following the assay procedure instructions.

### 2.13. RNA Extraction and Quantitative PCR

RNA was extracted from chondrocyte/scaffold composites by adding TRIzol (Life Technologies, USA). The cDNA was reverse-transcribed using Master Mix (Applied Biosystems, USA). cDNA (1 *μ*l) was amplified in a 20 *μ*l PCR mixture including SYBR Green Real-time PCR Master Mix-Plus (Applied Biosystems, USA) and gene-specific primers (Table 1) in accordance with the manufacturer's instructions. The reaction comprised an initial denaturation at 95°C for 2 min, 40 cycles with denaturation at 95°C for 15 s, and annealing and extension at 55°C for 15 s in each cycle and was performed using a StepOne Real-Time PCR System (Applied Biosystems, USA). Primer sequences were designed based on the published gene sequences (NCBI and PubMed). GAPDH was chosen as an endogenous control for the study. The relative gene expression profiles of various samples were normalized to the corresponding GAPDH and analyzed using the 2−ΔΔCT approach. Three replicates were made per sample.

### 2.14. Histology and Immunohistochemistry (IHC)

For the histological analysis, after concluding the 28 days of maturation, 2CI and 3CI implants (*n* = 3) were fixed in Bouin's solution for 14 h. Afterward, the samples were dehydrated gradually with acetones and embedded in paraffin. In all, 5 *μ*m thick sections were cut and stained using hematoxylin and eosin (H&E) in order to visualize the cell morphology, Masson's trichrome stain to observe the collagen fibers, and safranin O/fast green to detect the presence of sulfated proteoglycans.

The sections were also stained for the presence of collagen type II and collagen type I, using immunohistochemistry following a standard protocol. Mouse monoclonal primary antibodies (all from Abcam, Cambridge, UK) against collagen type II (1 : 500 dilution) and collagen type I (1 : 300 dilution) were incubated at 4°C overnight for immunostaining. Sections were incubated with a biotinylated goat antipolyvalent secondary antibody (1 : 200 dilution, ab64264, Abcam), followed by streptavidin peroxidase from the detection kit (Abcam), and developed with a mixture of 20 *μ*l 2.5% DAB chromogen and 1 ml DAB substrate from the detection kit (Abcam) for 10 min at room temperature. Finally, sections were counterstained with hematoxylin and viewed and also photographed using an Olympus AX70 microscope (Olympus, Tokyo, Japan).

### 2.15. Histological Evaluation

A modified Bern/O'Driscoll score was used to evaluate the *in vitro*-generated cartilaginous tissue which allows a relevant association with parameter characteristic of cartilage quality. It considers cell morphology, tissue integrity, proteoglycans, etc. The articular cartilage lesion was graded, and the defects were evaluated by three independent experts who were familiar with the histology of cartilage repair and were double-blinded to the groups. All scores used for calculating differences between groups were means of the independent evaluations.

### 2.16. Ethics Statement

The protocol involving research in animals was approved by the UANL School of Medicine and University Hospital Institutional Review Board (reference number: BI12-002), and the experiments were conducted following the Mexican standard for the treatment of experimental animals (Norma Oficial Mexicana 062-ZOO-1999).

## 3. Results

### 3.1. Isolation and Characterization of Ovine ADSCs

**Figure 1:**
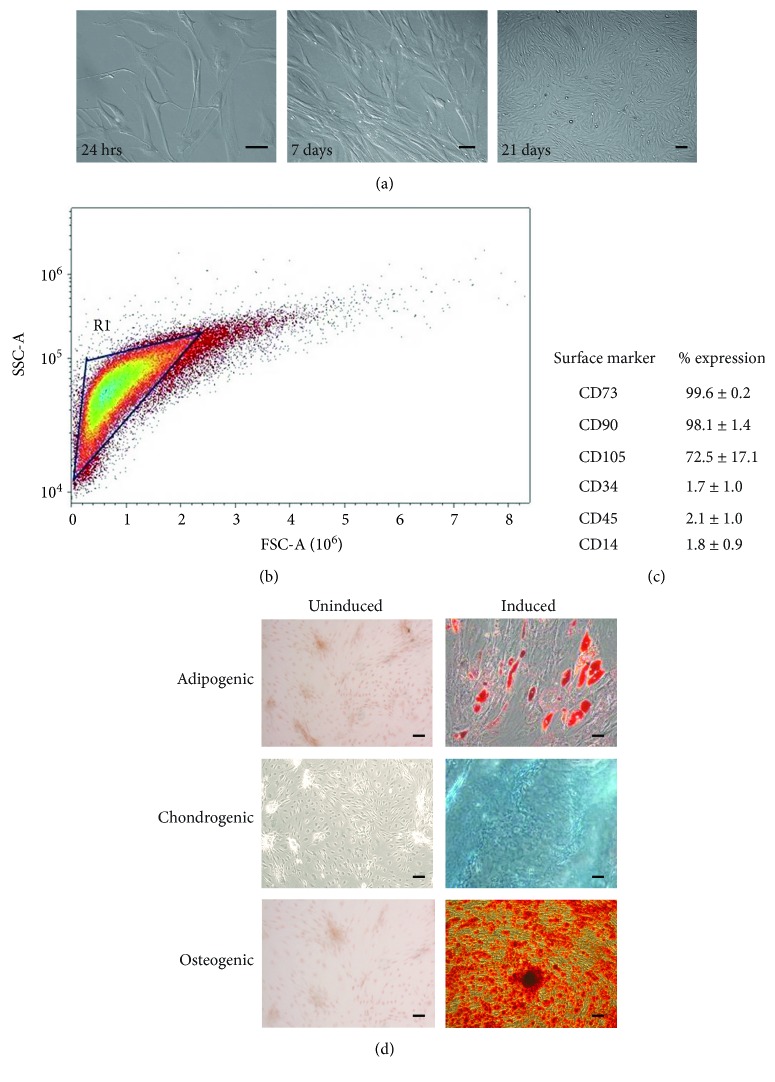
Isolation and characterization of ADSCs from the adipose tissue of *Ovis aries*. (a) Monolayer of adherent spindle-shaped fibroblastoid cells compatible with undifferentiated mesenchymal stem cells. Culture of the adherent cell population at 24 h postisolation. Cells cultured with the DMEM after 7 days. The cell culture reached confluence after 21 days (scale bar = 80 *μ*m). (b) Flow cytometry analyses are illustrated. FSC by SSC indicates a homogeneous population in size and granularity. (c) Surface marker expression: the data represent the mean percentage ± standard deviation of 3 independent experiments. (d) Trilineage differentiation of ADSCs into adipocytes, osteoblast, and chondrocytes is shown. Oil red staining of ADSCs induced for adipocyte differentiation. Alcian blue staining of ABMCs induced for chondrocyte differentiation. Osteoblast differentiation visualized using Alizarin red (scale bar = 80 *μ*m).

An adherent cell population was isolated by collagenase I digestion. Adherence to the plastic flask was observed after 24 h of culture, as shown by the presence of spindle-shaped cells (Figure 1(a)). During the passages, the cells become more flat-shaped and reached 80-90% confluence in approximately 21 days. After three passages, cultures were represented homogenously as typical morphology of fibroblast-like cells (Figure 1(a)). The characterization of cell-surface markers on isolated ADSCs was performed by flow cytometry from passage 3. As shown in Figure 1(b), FSC by SSC indicates a homogeneous population in size and granularity. To confirm the MSC phenotype, the expression of MSC- and HSC-specific cell-surface markers was analyzed. ADSCs were positive for mesenchymal antigens CD73 (99.6 ± 0.2%), CD90 (98.1 ± 1.4%), and CD105 (72.5 ± 17.1%) and negative for the expression of hematopoietic markers such as CD34 (1.7 ± 1.0%), CD45 (2.1 ± 1.0%), and CD14 (1.8 ± 0.9%); this data represents a homogeneous population of MSCs. To investigate the differentiation potential of the ovine ADSCs, cells were subjected to osteogenic, adipogenic, and chondrogenic differentiation (Figure 1(d)). Adipogenic differentiation was observed by the accumulation of cytoplasmic lipid vacuoles and oil red O staining. Chondrogenesis was verified by staining with Alcian blue, a blue signal corresponding to the matrix rich in proteoglycan, while osteogenic differentiation was evidenced by calcium deposition, stained by Alizarin red.

### 3.2. Mechanical Testing of the Alginate/Bovine Cartilage Matrix Scaffold

**Figure 2:**
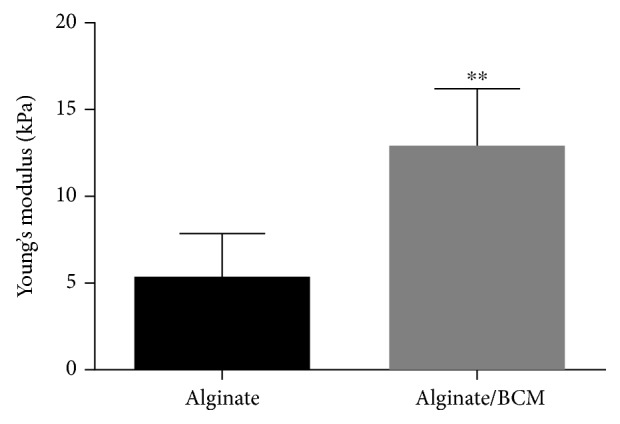
Young's modulus of 1.2% alginate scaffolds, crosslinked in CaCl_2_ and 1.2% alginate biofunctionalized with BCM. Data points represent the mean from *n* = 10 ± SEM. The samples show significantly different moduli (^∗∗^*p* < 0.05).

To evaluate whether the addition of the bovine cartilage matrix to alginate contributed to a better mechanical scaffold, the compression modulus was measured to the implant. The alginate scaffold exhibited an average Young's modulus of 5.2 kPa (Figure 2), while alginate/BCM reported 10.21 kPa. The biofunctionalization with BCM significantly increased the compressive modulus 2-fold over the alginate scaffold alone.

### 3.3. Cellular Distribution and Viability onto the Alginate/Bovine Cartilage Matrix Tridimensional Scaffold

**Figure 3:**
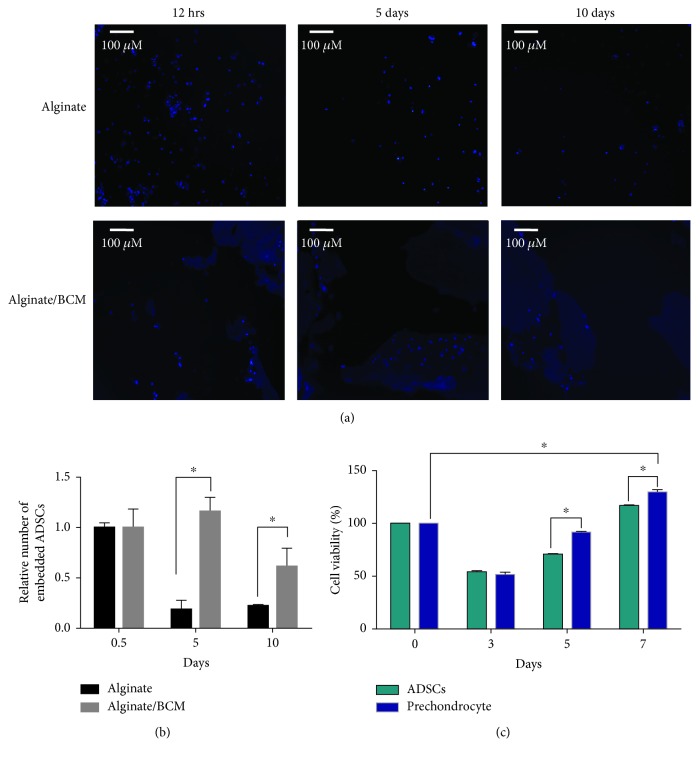
Cellular distribution and viability onto the alginate/bovine cartilage matrix tridimensional scaffold. (a) ADSCs retained in the scaffolds after 12 h and 5 and 10 days of cellularization, evaluated by fluorescence microscopy according to the number of nucleus stained with DAPI. (b) Quantitative image analysis: the data represent the average of 15 fields taken as 1.0, the number of cells at 12 h. (c) Alamar Blue cell viability assay of ADSCs and prechondrocytes in the biofunctionalized alginate scaffold at 0, 3, 5, and 7 days of culture (^∗^*p* < 0.05).

To analyze if the distribution of cells is influenced by the addition of BCM, histological sections of the scaffolds cellularized with ADSCs were analyzed by fluorescence microscopy. As shown in Figure 3(a) in the alginate/bovine cartilage matrix, as the implant matures (after 10 days of culture), the cells acquire a more orderly distribution, surrounding the BCM. On the other hand, the cells in the alginate scaffold came out from the implant as can be seen in Figure 3(a), which explains the drastic decrease in the relative number of embedded cells shown in the graph (Figure 3(b)).

Once the cellular diffusivity in the implant was demonstrated, we evaluated the ability of the scaffold to support the proliferation of the ADSCs and prechondrocytes in the three-dimensional system. Cell proliferation of ADSCs and prechondrocyte cells in the alginate/BCM scaffold was assessed using the Alamar Blue quantitative assay after 0, 3, 5, and 7 days of culture (Figure 3(c)). The spectrofluorometric data show that approximately 50% of the cells remain anchored to the scaffold within the first 3 days. As shown in Figure 3(c), the viability of prechondrocytes appeared significantly higher than that of the ADSCs at the 5^th^ and 7^th^ day (*p* < 0.05). These results indicate that the alginate/bovine cartilage matrix scaffold effectively supports cell proliferation, generating a suitable environment for prechondrocytes.

### 3.4. Secretion of IGF1 in Transduced ADSCs Cultured on the Alginate/Bovine Cartilage Matrix Scaffold

**Figure 4:**
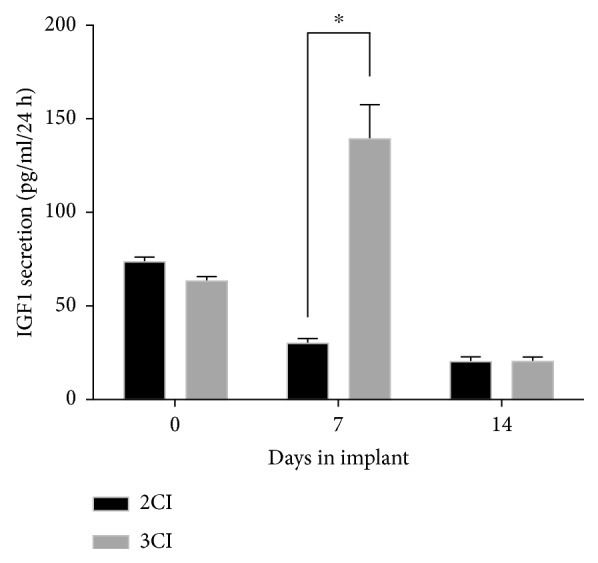
Secretion of IGF1 in ADSCs cultured on the alginate/bovine cartilage matrix scaffold. Secretion of the IGF1 protein to the culture medium in nontransduced (2CI) and transduced (3CI) ADSCs after 7 and 14 days in the 3D system. A significant difference was observed at day 7 of culture in the implant 3CI compared with 2CI (^∗^*p* < 0.05).

The ADSCs, as well as the prechondrocytes evidenced in the implant, had a proliferation rate with a similar trend. To assess the effect of IGF1 overexpression on the three-dimensional system, ADSCs transduced with the adenoviral vector Ad-IGF1 with an MOI of 100 (ADSCs-AdIGF1) and nontransduced ADSCs were seeded on an alginate/BCM scaffold and cultured in chondrogenic medium (3CI and 2CI groups, respectively). To assess the activity of Ad-IGF1, the presence of the IGF1 secreted into the culture medium was analyzed by ELISA. The 3CI group showed at the 7th day of culture a significant increase (5-fold) in the secretion of this protein compared to the 2CI group (*p* < 0.05) and a 2-fold increase compared to the baseline (time 0, *p* < 0.05) (Figure 4), indicating that the adenovirus was active and helped in overexpressing this protein. It should be noted that at day 14, in both groups 3CI and 2CI, the secretion of IGF1 dropped 2-fold compared to basal secretion prior to chemical induction into the chondrogenic lineage (ADSCs have an intrinsic basal expression of IGF1).

### 3.5. Microarchitecture of the 3CI and 2CI Implants

By scanning electron microscopy, the microstructure of the 3CI and 2CI design was analyzed.

**Figure 5:**
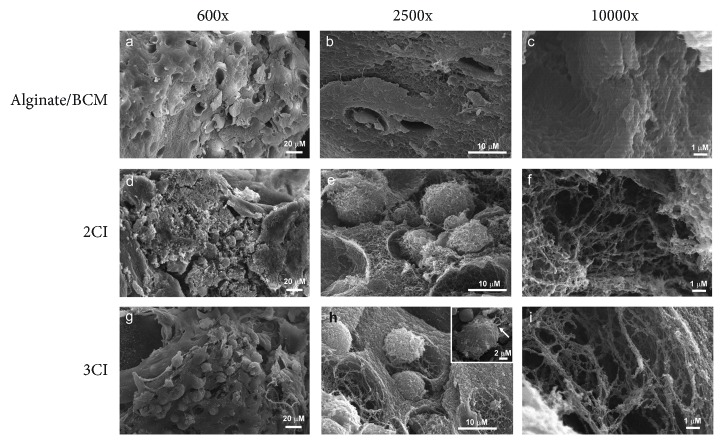
SEM micrographs of nontransduced (2CI) and transduced (3CI) ADSCs grown on the alginate/bovine cartilage matrix scaffold. (a–c) Micrographs showing the ECM of decellularized bovine cartilage. (d–f) Micrographs showing the longitudinal views of the 2CI implant. (e) High magnification revealed cell adhesion and chondrocyte-like morphology. (f) Matrix synthesis and fibers around the cells after 28 days of culture. (g–i) Longitudinal views of the 3CI implant show the same characteristics as 2CI and also highlight the formation of typical isogenous groups (g and h) and filopodia-like structures (inset in h) after 28 days of culture.

The alginate/BCM nonseeded scaffold showed a smooth surface with uniformly interconnected pore structures, as observed by SEM, and with a pore size width of about 100 *μ*m, allowing large spaces for cell growth (Figures 5(a)–5(c)). Furthermore, 2CI and 3CI groups were cultured for 28 days in chondrogenic medium for maturation. Both 2CI and 3CI showed homogeneous cell distribution throughout the scaffold (Figures 5(d) and 5(g), respectively). The cells appear embedded within the secreted ECM, and highly dense growth of the cells on the matrices (Figures 5(e) and (h)) allowed them to entwine to form a mesh (Figures 5(f) and 5(i)) with some long filopodia attached to the substrate (Figure 5(h), inset). This suggests that the average pore size and different material properties such as structure and surface chemistry of the scaffold provide a suitable environment for cell adhesion, proliferation, and growth.

### 3.6. Expression Analysis of Chondrogenic Differentiation Markers in the 3CI and 2CI Implants

**Figure 6:**
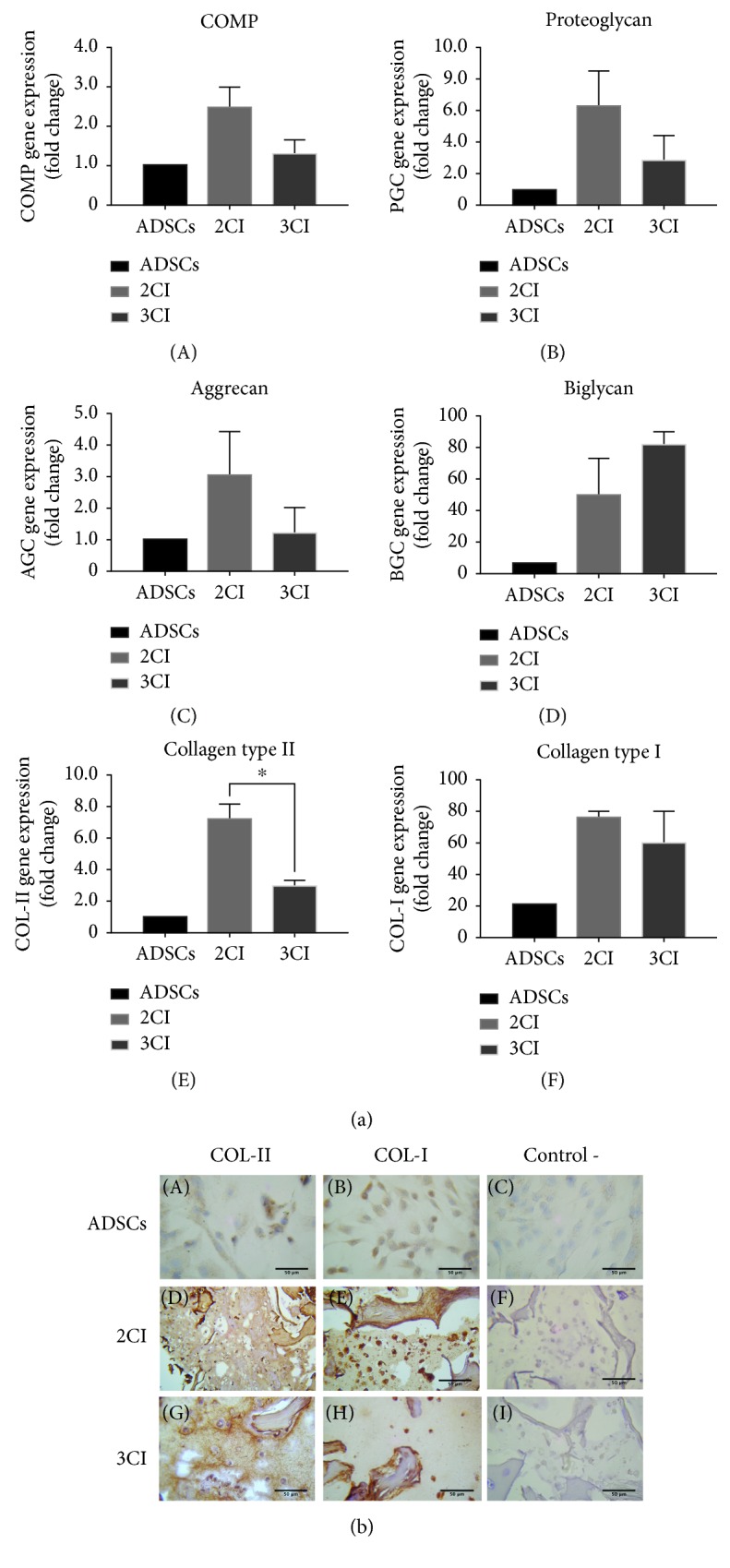
Expression analysis of chondrogenic differentiation markers in the 3CI and 2CI implants. (a) Relative gene expression of (A) COMP, (B) proteoglycan, (C) aggrecan, (D) biglycan, (E) collagen II, and (F) collagen I in ADSCs and in 2CI and 3CI groups. Data are reported as mean ± standard deviation (*n* = 3). A significant difference was observed for collagen II in the implant 2CI compared with 3CI (^∗^*p* < 0.05). (b) Representative images at day 28 for type II and type I collagen immunohistochemistry. ADSCs: adipose-derived mesenchymal stem cells cultured in a monolayer. 2CI and 3CI showed positive staining for type II collagen and strong signal for type I collagen as well. Negative controls were treated with PBS without primary antibodies. Scale bar = 50 *μ*m.

For chondrogenic implant maturation, 3CI and 2CI were cultured in chondrogenic medium for 28 days. For further assessment of the effect of IGF1 overexpression on the gene expression characteristics of the chondrocytes, mRNA levels of cartilage ECM components such as collagen II, collagen I, aggrecan, COMP, PGC, and BGC were detected by RT-qPCR. 3CI and 2CI cultured in the chondrogenic medium displayed a significantly higher expression for all the genes analyzed than the ADSC control group, showing an expression profile according to a mature chondral tissue. Regarding the chondrogenic gene expression markers COMP, PGC, and AGC, they showed after 28 days of culture an increasing trend in 2CI compared to 3CI; however, the data did not show statistically significant differences (Figure 6(a)). Besides, biglycan gene expression, a known marker of functionally mature chondrocytes, was slightly increased when IGF1 was overexpressed (3CI) (Figure 6(a), D). Surprisingly, the mRNA expression of type II collagen was 2-fold higher in the 2CI group compared to the 3CI group (Figure 6(a), E). Type I collagen is considered a fibrotic marker of hyaline cartilage. After 28 days of scaffold culture, COL1A1 mRNA levels are slightly upregulated by 2-fold and 1.5-fold in 2CI and 3CI, respectively, compared with undifferentiated ADSCs cultivated in monolayer culture (Figure 5(f)). No statistically significant differences were detected among the groups at 28 days. These results suggest that at a late stage (28 days) at the transcriptional level, the overexpression of IGF1 mediated by an adenoviral vector offers no advantage.

An important issue in the regeneration of hyaline cartilage is the imbalance between type II collagen production and type I collagen expression, which promotes the development of fibrosis; thereby, the distribution of the Col-II and Col-I proteins was analyzed by immunohistochemistry. An IHC assay was performed to visualize collagen distribution in the 3CI and 2CI groups after 28 days in chondrogenic medium.  Figure 6(b) shows that in 3CI, type II collagen staining was more marked with a preferential localization towards the periphery of the rounded cells. In contrast, the type II collagen signal in 2CI predominates in the neoformed matrix. For type I collagen, the expression in the 3CI and 2CI groups has a comparable intensity between them, showing a scarce network between the cells with a pronounced distribution towards the neoformed extracellular matrix.

In conclusion, the 3CI design shows a better distribution and balance of type I and II collagens compared to 2CI.

### 3.7. Histological Evaluation of the 3CI and 2CI Implants

**Figure 7:**
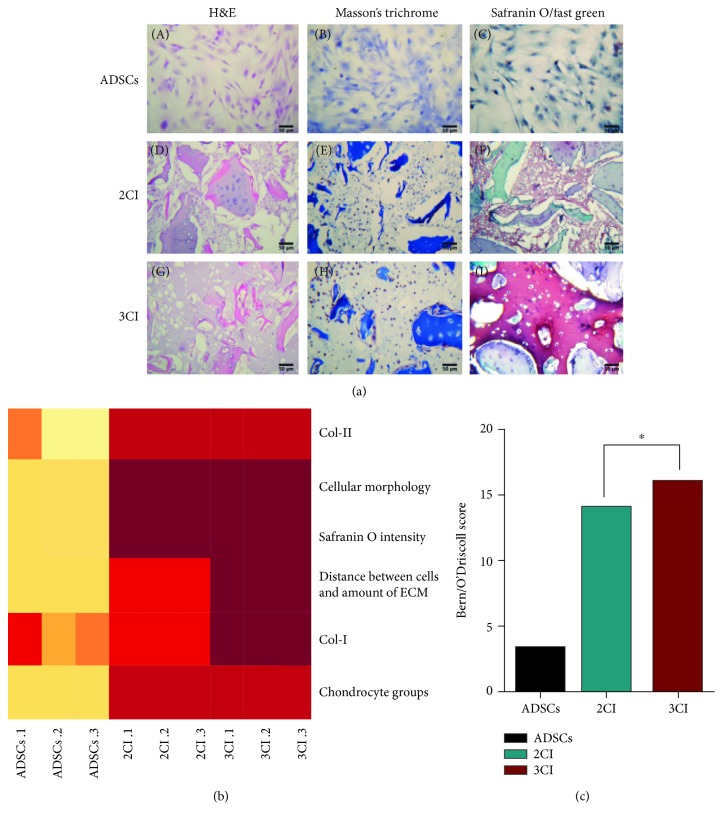
Histological evaluation of the 3CI and 2CI implants by the Bern/O'Driscoll scale. (a) Histological evaluation of 2CI and 3CI groups after 28 days of culture. (A–C) Adipose-derived mesenchymal stem cells cultured in a monolayer as a negative control. 2CI and 3CI showed the typical rounded morphology of chondrocytes (D, G), as well as strongly positive staining of bovine cartilage fragments for Masson's trichrome, and also exhibited neomatrix formation with chondrocyte-like appearances (E, H). Interestingly, sections stained with safranin O to detect proteoglycan (in red) (F, I) show for 3CI a dense neomatrix formation that is strongly stained (I) (scale bar: 50 *μ*m). (b) Heat map generated by unsupervised clustering analysis programming in R. The intensity of color varies according to a given value criterion, and similar color patterns are grouped together. Strong colors (red) show a greater similarity to the natural cartilage tissue. (c) Histological analysis by the Bern/O'Driscoll score. The summation of parameters evaluated for each experimental group show that 3CI was more similar to natural cartilage tissue in comparison with 2CI (^∗^*p* < 0.05) as evidenced by the Wilcoxon test for nonparametric data.

By H&E staining, the cellularity in the implant was evaluated as well as the presence of the neoformed cartilage matrix as visualized in acidophilic staining. The 3CI design evidences a dense production of the neoformed matrix, and a low cellularity was also observed. In contrast, in the 2CI group, although presenting a higher number of cells per field, the neoformed matrix is scarce and dispersed (Figure 7(a)).

Masson's trichrome staining demonstrated the presence and distribution of collagen fibers. Both groups, 3CI and 2CI, showed very similar distribution patterns with poor staining of collagen fibers in the neoformed matrix. Interestingly, overwhelming differences between the 3CI and 2CI groups were observed with regard to the production of sulfated proteoglycans (in red) by safranin O/fast green staining. The 3CI group showed an enhanced and extensive staining for proteoglycans within the intercellular space (red staining), while the surrounding ECM stained reddish blue due to the dual presence of safranin-positive proteoglycan and fast green-stained collagen. The presence of large amounts of proteoglycans associated with a small number of cells matches with the characteristics of hyaline cartilage tissue. On the other hand, the 2CI group showed a moderate red staining for safranin with a scattered intercellular space and a disordered and clustered arrangement of the chondrocytes.

### 3.8. Histological Grading of the 3CI and 2CI Implants by the Bern/O'Driscoll Score

The ultimate goal of tissue engineering is that it can be applied in replacement therapy to achieve the restructuring of tissue architecture. There are different histological scales for assessment to determine the nature of the newly formed tissue *in vitro*; for this analysis, the Bern scale was used for evaluation, along with some parameters of the O'Driscoll scale and further observations of immunohistochemistry for Col-I and Col-II. The score for each parameter evaluated, ranging from 0 to 3, with 0 being the worst result and 3 the most optimal result, indicated that it was similar to hyaline cartilage.

A heat map with the numerical values evaluated was generated to display the distribution of variables; the red color denotes a higher score in the evaluation scale and light colors denote a lower score. It should be noted that the absence of Col-I, as well as the cell distance and amount of matrix in the 3CI design, recorded the highest score. Out of a total of 18 points, the 3CI group obtained 16, the 2CI group 14, and the control basal culture 3.33. The sum of the evaluated parameters was plotted, and the data were analyzed by the Wilcoxon test for nonparametric variables; results were statistically significant for the 3CI group compared with the 2CI group (*p* < 0.05), which means that 3CI has more features similar to those of a native tissue (Figures 7(b) and 7(c)).

## 4. Discussion

Development of novel therapies for the treatment of osteoarthritis is a major challenge for tissue engineering and regenerative medicine. A growing clinical demand for suitable grafts for damage repair in joint surgery has promoted the development of new designs to fully repair in terms of covering the defect with high-quality engineered cartilage constructs. The results of this study establish the design of an implant constituted by ADSCs transduced with an IGF1 adenoviral vector and embedded in a combined scaffold made of an alginate/bovine cartilage matrix to repair focal articular defects.

Cell-based therapeutic approaches in tissue engineering and regenerative medicine have highlighted the need for the utilization of abundant and undifferentiated progenitor cells. ADSCs are highly proliferative and multipotent, representing a great promise in regenerative medicine. This study used ADSCs, which allows obtaining stem cells for autologous transplantation. As shown by FACS analysis, a homogenous adherent cell population was isolated by collagenase I digestion (Figure 1(c)); the isolated cells express mesenchymal antigens CD73, CD90, and CD105 and also were negative for HSC markers CD34, CD45, and CD14 (Figure 1(d)). Similar to previous studies, the expression profile of surface markers correlates with cell-based isolation with collagenase I digestion [27, 28]. Nevertheless, we found a discrepancy regarding CD90 expression, which according to Maddox et al., its expression in ADSCs is negative. Apparently, the observed difference comes from the collagenase II digestion of the adipose tissue, which drives the isolation of a different stem cell subpopulation [29].

Previous studies have used alginate as a hydrogel scaffolding system. These systems can be formed at physiological conditions with high water content [30], which facilitates the immobilization of cells within the hydrogel; however, inferior mechanical properties compared with those of natural cartilage and a sparse interaction with the cells are issues to be overcome.

The biomimetic characteristics of the scaffolds used in tissue engineering are the key for the proper regeneration of joint cartilage. An ideal scaffold for cartilage repair should promote the synthesis of the cartilage matrix. Since ECM functions as a source for growth factors and cytokines that modulate the state of cellular activation [31], ECM-based materials harvested from various tissue sources, such as skin, blood vessels, and heart valves, have been used in the construction of scaffolds for the repair of many tissues [32–34]. Specifically, the ECM of hyaline cartilage has substantial amounts of collagen and proteoglycans and, in a relevant manner in terms of cartilage tissue, also has antiangiogenic effects [35–37]. The ECM derived from the cartilage of cows, pigs, and humans has been developed and used for the regeneration of cartilage, to provide a natural microenvironment to support the adherence, proliferation, and differentiation of stem cells into chondrocytes.

These data in the literature support the observation that the addition of BCM to alginate helps keep the ADSCs embedded in the scaffold (Figure 3(b)) and facilitates the distribution of cells along the scaffold in areas where the particles of the BCM are located (Figure 3(a)). Furthermore, the distribution of cells in proximity to the BCM particles also offers a suitable microenvironment for the viability of prechondrocytes. As we could identify the cells embedded in the alginate/BCM scaffold, they showed a marked recovery in metabolic cell activity between 3 and 7 days. This observation demonstrated that the microenvironment is not toxic to cells. Interestingly, the metabolic activity of the prechondrocytes showed a better recovery compared to that of the ADSCs, which confirms that the scaffold enriched with BCM supports chondrogenic properties (Figure 3(c)).

Previous studies have used BCM as an efficient chondrogenic inducer [38–40], and although its benefits in terms of extracellular matrix production have been addressed, mechanical properties have scarcely been analyzed. Young's modulus defines the ability of the scaffold to resist deformation when subjected to stretching or compression forces. In this study, it is demonstrated that in a hydrogel scaffolding system based on alginate, the incorporation of BCM with a controlled particle size improves the elastic properties of the final scaffold, since the alginate/BCM scaffold increases the magnitude of Young's modulus by 2 times compared with alginate by itself (Figure 2).

The distribution and pore size on the scaffold have an effect on several cell responses, as well as on adequate tissue organization, cell adhesion, vascularization, and finally tissue regeneration [41].

As a source for a natural cartilaginous matrix, bovine knee was chosen since it allows a high-yield recovery of a material rich in collagen fibers; proteoglycans are also highly porous. By decellularization, we attempt to remove chondrocytes but also increase the porosity of the BCM and therefore the final scaffold. Lin et al. showed that pore sizes between 250 and 500 *μ*m in scaffolds are suitable for chondrocyte proliferation and adequate ECM secretion [42]. Certainly, the presence of macropores (>50 *μ*m) is important for the 3D scaffold to promote cell migration [43]; however, it has been reported that the presence of micropores also promotes cell-cell interaction and mass transport, which improve tissue formation [44, 45]. According to SEM analysis, the combined use of the bovine cartilage matrix and 1.2% alginate hydrogel allowed pore formation ranging from 50 *μ*m to 100 *μ*m (Figure 5), which impacted positively on cell colonization of the scaffold (Figures 5(d) and 5(g)) and the subsequent structuring of a dense extracellular matrix (Figures 5(e) and 5(h)).

The implant based on alginate and the matrix of bovine cartilage characterized in this study was analyzed with respect to its mechanical properties, its ability to sustain growth, and also its chondroinductive ability when the cell component overexpresses IGF1. The isolated ADSCs were initially expanded in the monolayer, transduced with the adenoviral vector, and induced with chondrogenic medium until reaching the prechondrocyte stage. They were then embedded in the alginate/BCM scaffold, which allowed cell infiltration (Figure 3(a)) and provided a microenvironment capable of sustaining cell growth (Figures 5(d) and 5(g)).

By adding growth factors to the system, cartilage maturation can be induced, resulting in cartilage regeneration [46]. ADSCs can differentiate into chondrocytes under specific culture conditions. A variety of biological factors, such as TGF-*β*1, IGF1, and FGF, are often used to induce the chondrogenic differentiation of several adult stem cells. IGF1 is one of the widely used growth factors due to its effect on cell proliferation and also differentiation into articular chondrocytes during growth plate development [47].

The effects of IGF1 on chondrogenic differentiation are controversial in recent years, since authors like Kawamura et al. reported that adenoviral expression of IGF1 in human MSCs inhibited collagen II expression and did not promote chondrogenesis [48]. Ochiai et al. even reported that its overexpression can induce hypertrophic differentiation and mineralization [49]. On the other hand, Frisch et al. demonstrated that MSC treatment with the IGFI vector increased the proliferation rate, matrix production, and chondrogenic differentiation [50]. Likewise, Madry et al. in a nonviral delivery of IGF1 into rabbit articular chondrocytes reported the enhanced production of glycosaminoglycan over a temporal kinetic [51].

Although we report, at 28 days of maturation, an increase in the expression of the transcripts of COMP, proteoglycan, aggrecan, and Col-II in response to the overexpression of IGF1 [26], in the three-dimensional system (alginate/BCM), the response at the transcriptional level did not show significant differences between 2CI (untransduced ADSCs) and 3CI (ADSCs transduced with Ad IGF1) implants (Figure 6(a)). It should be noted that this assay was only used on day 28 of implant maturation, so it is possible that the most obvious differences in the expression of these transcripts occurred at an earlier time during the differentiation process.

According to our strategy, overexpression of IGF1 in 3CI is only maintained for 7 days (Figure 4). Due to this, the observed effect at the transcriptional level after 28 days of culture is not appreciable; nonetheless, our data demonstrated that this design has at the protein level a good balance between Col-I and Col-II expression, which results in a structure similar to that of the native cartilage (Figure 6(b)) as well as enhanced expression of proteoglycans (Figure 7(a)) whose influence on the mechanical features is well known [52]. Furthermore, it is shown that the extracellular matrix is dense and well-structured and lacks fibrocartilage compared to the group not transduced with Ad-IGF1 (2CI). Therefore, controlled delivery of biomolecules such as IGF1 during the regenerative process is important to avoid adverse effects.

The chondroinductive effect of the bovine cartilage matrix together with the temporary overexpression of IGF1 had a positive impact mainly on the production of proteoglycans but also on the balance between Col-II and Col-I. Moreover, IGF1 showed a significant effect on the production of PGs as observed by staining with safranin O/fast green, giving a deep red color and a denser matrix compared to the adenovirus-lacking 2CI (Figure 7(a)).

The heat map highlights two significantly different parameters between 2CI and 3CI: “distance between cells and amount of matrix,” and staining for Col-I with the 3CI implant shows a better behavior. Regarding the distance between cells and the amount of matrix, it is indicative that the embedded chondrocytes are able to depolymerize the ECM to widen the gaps, where the chondrocytes, depending on their metabolic activity, are able to secrete a new intercellular matrix resulting in extensive distance between the chondrocytes as evidenced in the 3CI group (Figure 7(b)).

Similar to this work, Diekman et al. [53] used a scaffolding system, BCM, and alginate separately, and the induction towards chondrogenesis of ADSCs was done by chemical induction. Immunohistochemical results showed that both systems induce a chondrocytic phenotype with large amounts of Col-II, as well as Col-I, even in the absence of Col-X. The design presented here, based on the combined use of alginate and BCM, in addition to the overexpression of IGF1 (3CI), influences the balance between Col-II and Col-I. On the other hand, Chang et al. characterized a scaffold system composed of uncontrolled size fragments of cartilage and synovial MSCs induced towards chondrogenesis with medium containing dexamethasone, TGF-*β*3, and BMP-2. In this system, the gene expression showed a significant increase in Col-II on day 28; however, the density of the extracellular matrix observed was poor.

## 5. Conclusions

The histological studies evaluated by the Bern/O'Driscoll scale showed that in the 3CI group, a good balance between Col-II and Col-I is generated and the extracellular matrix is dense with a high content of proteoglycans which subsequently favors the mechanical properties of the implant. For this reason, the 3CI design has a histological type that is closer to the type that is desirable for the regeneration of cartilage, and the 3CI group (16/18) was significantly higher (*p* < 0.05) than the 2CI group (14/18), representing high-quality engineering cartilage suitable for *in vivo* tests.

## Figures and Tables

**Table 1:** 

Gene	Ref. no.	Sequence 5′-3′	Amplicon (bp)
Col-I	FJ200442.1	Fw: GGTGACAGGAAGTCCCAGAA	167
		Rv: CCATCGTAGGTGACGCTGTA	
Col-II	X02420.1	Fw: CTACTGGATTGACCCCAACC	211
		Rv: TGTCCTTGCTCTTGCTGATG	
Aggrecan (AGC)	NM_173981.2^∗^	Fw: CAGAGTTCAGTGGGACAGCA	189
		Rv: AGACACCCAGCTCTCCTGAA	
Proteoglycan (PGC)	NM_174288.1^∗^	Fw: TGCTGTGATTGCCTCTTTTG	169
		Rv: CCAAAACCCGTAGTTCCTGA	
Biglycan (BGC)	BT021201.1^∗^	Fw: ACCTCCCTGAGACCCTCAAT	184
		Rv: TTGTTGTCCAAGTGCAGCTC	
Cartilage oligomeric matrix protein (COMP)	X74326.1^∗^	Fw: ATGCGGACAAGGTGGTAGAC	153
		Rv: TCTCCATACCCTGGTTGAGC	
GAPDH	NM_001190390.1	Fw: CCATCACCATCTTCCAGGAGCG	481
		Rv: AAGGCCATGCCAGTGAGCTTC	

^∗^The sequence was designed based on the *Bos taurus* genome since the *Ovis aries* genome was not assembled for this gene.

## Data Availability

The data used to support the findings of this study are included within the article.
